# Comparison of the Rowe–Kahn Model of Successful Aging With Self-rated Health and Life Satisfaction: The West of Scotland Twenty-07 Prospective Cohort Study

**DOI:** 10.1093/geront/gnv054

**Published:** 2016-03-12

**Authors:** Elise Whitley, Frank Popham, Michaela Benzeval

**Affiliations:** ^1^MRC/CSO Social and Public Health Sciences Unit, University of Glasgow, UK.; ^2^Institute for Social and Economic Research, University of Essex, Colchester, UK.

**Keywords:** Successful aging, Self-rated health, Life satisfaction, Cohort

## Abstract

**Purpose of the Study:**

With increasing longevity in industrialized populations, there is growing interest in what defines “successful aging” (SA). Various SA measures have been proposed but no consensus has been reached and many have been criticized for not representing the views and priorities of older people. We consider whether the Rowe–Kahn SA model captures older individual’s perceptions of their own health and aging.

**Methods:**

Using two cohorts of 886 and 483 men and women from the West of Scotland Twenty-07 Study, aged around 57 and 76, respectively, we explored associations between Rowe–Kahn SA dimensions (absence of disease/disability; good physical/cognitive functioning; good interpersonal/productive social engagement) and four aspects of self-rated health and satisfaction (current general health; health for age; satisfaction with health; satisfaction with life).

**Results:**

Respondents’ self-rated health and satisfaction was generally good but few had all six Rowe–Kahn dimensions positive, the conventional definition of SA. All individual positive SA dimensions were associated with better self-rated health and satisfaction. This was consistent across age, gender, manual/nonmanual occupations, and personality. The prevalence of good self-rated health and satisfaction increased with increasing numbers of positive SA dimensions.

**Implications:**

The Rowe–Kahn model provides a functional definition of SA. Future work on ageing should include all Rowe–Kahn dimensions and consider SA as a continuum.

Industrialized populations are aging (Christensen, Doblhammer, Rau, & Vaupel, 2009) and medical advances mean individuals are living for longer, with correspondingly increasing susceptibility to disease and disability in later life. There is therefore substantial interest in what constitutes “successful aging” (SA; Bowling, 2007; Bowling & Dieppe, 2005; Katz & Calasanti, 2015; Kivimaki & Ferrie, 2011; Martin et al., 2015). SA is an important goal for health and economic policies (Bloom et al., 2015; Commission of the European Communities, 2009; United Nations, 2002) and effective measurement is vital for understanding the challenges, informing potential interventions, and monitoring progress toward its achievement. Many SA definitions focus on absence of disease and disability but do not necessarily reflect older people’s priorities. Our aim is to explore a broader, multi-dimensional SA definition, to examine its patterning in the general population, and to consider the extent to which it captures individual’s perceptions of their own health and aging.

While many scales and tools have been developed to objectively define disease or disability, “health” is a more subjective concept. Clinical and lay opinions differ (Blaxter, 2004), and definitions are culturally, socially, and context specific, for example, varying by age, gender, socioeconomic status (SES), and personality (Blaxter, 2004; Bryant et al., 2014; Martinson & Berridge, 2015). Additionally, health and disease are not simply opposite states but are more realistically viewed as part of a continuum. Many specific SA definitions have been proposed (Cosco, Prina, Perales, Stephan, & Brayne, 2013a; Depp & Jeste, 2006) but no consensus has been reached and more recent work has given increasing emphasis to the views of older people themselves (Cosco, Prina, Perales, Stephan, & Brayne, 2013b; Ferri, James, & Pruchno, 2009; Jopp et al., 2015; Phelan, Anderson, Lacroix, & Larson, 2004; Reichstadt, Sengupta, Depp, Palinkas, & Jeste, 2010; Tate, Lah, & Cuddy, 2003). While most researcher-led definitions include longevity, absence of disease and disability, and good functioning (Cosco et al., 2013a; Depp & Jeste, 2006), older people prioritize social engagement, well-being, and personal resources, such as independence and acceptance.(Cosco et al., 2013b) In addition, evidence suggests that many older people who consider themselves to be aging successfully do not meet researcher-defined SA criteria (McLaughlin, Jette, & Connell, 2012; Montross et al., 2006; Strawbridge, Wallhagen, & Cohen, 2002; von Faber et al., 2001; Young, Frick, & Phelan 2009). SA measures based simply on longevity or clinical outcomes may therefore fail to capture the complete aging experience of older people, many of whom demonstrate considerable resilience in the face of physical and cognitive decline (Manning, Carr, & Kail, 2014), and who may engage in compensatory strategies to maintain their preferred lifestyles (Glass, 2003; Martin et al., 2015).

In light of these findings, the best measures of SA from the older person’s perspective may be self-rated (e.g., health or satisfaction with health and life, particularly relative to age). The subjective nature of these measures allows respondents to rate their health and satisfaction in the context of their individual beliefs, priorities, experiences, and circumstances. Moreover, self-rated health is a widely used health indicator and has been shown to predict morbidity and mortality (Jylha, 2009). However, it is also recognized that self-rated health measures, in isolation, have limitations. For example, a recent comparison of self-rated health with SF-36 health dimensions(Au & Johnston, 2014) found that vitality and, at older ages, physical functioning were strongly associated with self-rated health, while associations with other dimensions, particularly social aspects, were weak, suggesting that self-rated health alone may not fully represent all aspects of health. Self-rated measures are also known to be context specific. For example, women versus men, older versus younger individuals, and those with lower versus higher socioeconomic position have been shown to prioritise different aspects of health and to give different responses to questions about self-rated health (Blaxter, 1990; Eriksson, Unden, & Elofsson, 2001) Similarly, individuals with greater negative effect are more likely to give pessimistic reports of their health (Benyamini, Idler, Leventhal, & Leventhal, 2000; Blaxter, 1990, 2004; Kraus, Adler, & Chen, 2013) In addition, at a broader level, self-rated health has been shown to vary between cultures and countries (Jylha, 2009; Mitchell, 2005) and over time.(Mitchell, 2005) These variations present a particular challenge in the context of SA as older people’s self-rated measures are highly dependent on their previous experiences, expectations, and current (objective) health status and circumstances. For example, older individuals with better life-satisfaction have been shown to place greater value on mental, physical, and social activity as they age (Tate et al., 2003) while those in worse health are more likely to regard morbidity and dependency as an inevitable consequence of ageing (Sarkisian, Hays, & Mangione, 2002). Paradoxically, therefore, older individuals in worse health, with lower expectations, may actually consider themselves to be aging better as their experience matches their expectations. It is therefore important that SA measures incorporate the dimensions of SA (clinical and nonclinical) that are important to older people, but also that these are measured objectively in ways that are comparable across individuals and context and are not, therefore, grounded in different subjective expectations.

The majority of previous SA models have focussed purely on clinical outcomes, although a few have also encompassed nonclinical aspects (Bowling & Iliffe, 2006, 2011; Young, Fan, Parrish, & Frick, 2009). The most widely used model of this type was proposed by Rowe and Kahn (1997) and incorporates three dimensions: avoidance of disease and disability; maintenance of good physical and cognitive function; and good social engagement, both interpersonal (contacts and transactions with others) and productive (engagement in activities of value to society). The inclusion of social engagement is important as it moves the SA model from simply measuring potential (no disease/disability, good functioning) to encompass activity and autonomy. This is consistent with broader disability frameworks, both hierarchical (e.g., Nagi’s Disablement Model; Verbrugge & Jette, 1994) and multidirectional (e.g., WHO’s International Classification of Functioning, Disability and Health [ICF]; World Health Organization, 2001), which consider activity and participation in addition to pathology and functioning. However, it has been suggested that the Rowe–Kahn model does not adequately reflect the views of older people (Bowling & Iliffe, 2006, 2011; Ferri et al., 2009; Montross et al., 2006; Phelan et al., 2004; Strawbridge et al., 2002; Young, Fan, et al., 2009; Young, Frick, et al., 2009) We have therefore compared the Rowe–Kahn model with four established measures of self-rated health and life satisfaction in two cohorts of older men and women pre- and post-retirement age. In particular, we have included self-rated health *for age*, encouraging respondents to assess their health relative to their personal experiences, feelings, and expectations regarding the aging process.

The current analyses aim to address four main research questions. First, to what extent do the Rowe–Kahn dimensions agree with self-rated health and overall satisfaction? Second, do these results vary according to population subgroups that have been shown to vary systematically in the importance they attach to different dimensions of health (Blaxter, 1990, 2004), for example, by gender, age, SES, and personality? Third, is there an interplay between SA dimensions, either hierarchical (e.g., recognizing that disease may reduce physical functioning, resulting in poor social engagement (Verbrugge & Jette, 1994) or multidirectional (e.g., acknowledging the potential feedback loop whereby poor social function may also impact negatively on physical functioning, thereby increasing disability(World Health Organization, 2001)? Finally, given the continuum between poor and good health and previous evidence demonstrating that many older people who consider themselves to be aging well fail to succeed in all six Rowe–Kahn dimensions (McLaughlin, Connell, Heeringa, Li, & Roberts, 2010; McLaughlin et al., 2012; Montross et al., 2006; Strawbridge et al., 2002), we consider whether a continuum based on the number of successful Rowe–Kahn dimensions offers a more realistic measure of SA than the traditional binary measure.

## Design and Methods

The West of Scotland Twenty-07 study is a population-based multiple-cohort study (Benzeval et al., 2009), following three age-cohorts of men and women in the West of Scotland born around 1932, 1952, and 1972. Baseline interviews were conducted in 1987/1988, when the three cohorts were approximately 55, 35, and 15 years old. Respondents were representative of the population of the sampled area (Der, 1998). There were four follow-up waves in 1990/1992, 1995/1997, 2000/2004, and 2007/2008. Ethics approval was gained for each wave from the National Health Service and/or Glasgow University Ethics Committees. Current analyses are based on the final data collection wave in the two oldest cohorts.

### SA Dimensions

Details of the assessment of each Rowe–Kahn dimension (Rowe & Kahn, 1997) are presented in Table 1. Absence of disease was determined consistently in both cohorts using Royal College of General Practitioners codes, with respondents free from seven of the most common age-associated chronic diseases (listed in table) scoring positively. All other dimensions were considered separately by cohort, with “positive” dimensions based on the “best” cohort-specific (approximate) tertile. This approach acknowledges the natural age-related changes in disability and functioning (Weir, Meisner, & Baker, 2010) between two cohorts differing in age by 20 years, and allows for differences in social engagement between working-age and post-retirement cohorts. Disability was assessed using Office of Population Censuses and Surveys disability scores (Martin, Meltzer, & Elliot 1988). Good physical functioning was defined as 3+ of: above cohort-median grip strength or FEV_1_, below cohort-median systolic blood pressure or pulse. Cognitive function was based on Alice Heim 4 test of General Intelligence (Part 1; Heim, 1970). Good interpersonal engagement in both cohorts was defined as 3+ of: living with spouse/partner, recent contact with family/friends, attendance at clubs/classes. Productive engagement was based on work, training, volunteering, childcare, supporting others, and group memberships, with a higher cut-off in the 1952 (3+ activities) versus the 1932 (2+ activities) cohort reflecting higher employment rates in this group.

**Table 1.** Definition and Prevalence of Rowe and Kahn Successful Aging Dimensions Assessed at Final Wave of Data Collection in 1932 and 1952 Cohort Members

	Definition of positive successful aging dimension	*n* (%) positive dimension among those alive and interviewed at final wave	
		1952 cohort (*N* = 886)	1932 cohort (*N* = 483)
Disease and disability			
Absence of chronic disease	No coronary heart disease, stroke, chronic obstructive pulmonary disease, cancer (excluding skin), diabetes, Parkinson’s, or serious mental health problems^a^	719 (81.2)	280 (58.0)
No disability	Lowest (approximate) tertile of Office of Population Censuses and Surveys disability score (1952 cohort: 0; 1932 cohort: ≤0.5)	397 (44.8)	195 (40.3)
Functioning			
Good physical functioning	3+ out of 4 of height-standardized sex-specific grip strength and FEV_1_ above median and systolic blood pressure and pulse below median within each cohort	294 (33.2)	166 (34.4)
Good cognitive functioning	Part 1 of the Alice Heim 4 test of General Intelligence score in top tertile within each cohort (1952: 41+; 1932: 31+)	291 (32.8)	166 (34.4)
Social engagement			
Good interpersonal social engagement	Most engaged tertile based on: living with spouse/ partner, direct contact with family/friends in last 4 weeks, regular attendance at education/arts, social or sports club/class (3+ positive responses for both cohorts)	239 (27.0)	129 (26.7)
Good productive social engagement	Most engaged tertile based on: paid work/training, voluntary work, childcare, support to another, member of political/environmental, community, or church/charity group (1952: 3+ positive responses; 1932: 2+ positive responses)	174 (19.6)	120 (24.8)
Number of positive successful aging dimensions			
0		62 (7.0)	57 (11.8)
1		161 (18.2)	118 (24.4)
2		256 (28.9)	116 (24.0)
3		233 (26.3)	101 (20.9)
4		133 (15.0)	61 (12.6)
5		36 (4.1)	21 (4.4)
6		5 (0.6)	9 (1.9)

^a^Royal College of General Practitioners Classification and Analysis of General Practice Data codes: 0400-0540 (excluding 0455), 0720, 1000, 1015–1030, 1205, 1315, 1940–1950, 2100–2115, 2420, 2490–2510.

### Self-rated Health

In order to investigate lay perceptions of “successful ageing” we employed four different global scales that varied in wording or response items in ways that may systematically influence responses (Bowling, 2005; Eriksson et al., 2001). We included three health-specific scales. These focused on: (a) recent health, which is likely to prompt a response related to current experiences (“Over the last 12 months would you say that your health on the whole has been ...?” excellent/good (which we considered to be favorable) vs fair/poor); (b) health compared to people of same age, which has been shown to improve reporting of health at older ages as people make a relative comparison (Eriksson et al., 2001; “Would you say that for someone of your age your health in general is ...?” excellent/good [favorable] vs fair/poor); and (c) satisfaction with health, which may capture attitudes and coping strategies in addition to limitations to physical health (“Overall how do you feel about your health as it is now?” assessed on a seven-point scale: three most positive responses [favorable] vs neutral/three most negative responses). Finally, we included an overall measure of life satisfaction which asked respondents to evaluate their life as a whole rather than specifically focusing on health (“Overall how do you feel about your life as it is now?” assessed on a seven-point scale: three most positive responses [favorable] vs neutral/three most negative responses). Across these measures we hope to capture a range of dimensions of satisfaction with ageing, both in general and health related, overall compared with recent, and relative to others of own age.

### Statistical Methods

We calculated prevalences (95% confidence intervals) of favorable self-rated health and satisfaction (Muller & MacLehose, 2014) and explored the extent of agreement between the Rowe–Kahn dimensions and self-rated measures by considering the prevalence of favorable self-rated health and satisfaction separately according to each positive SA dimension. We used logistic regression models to establish whether these associations differed according to subgroups defined by age, gender, SES, and personality by performing analyses separately for: 1952 versus 1932 cohorts (aged around 57 and 76, respectively); men versus women; respondents with manual versus nonmanual occupations; and respondents with high versus low negative affect. Negative affect was measured using a general dimension of distress and unpleasurable engagement assessed using the trait version of the positive and negative affect scale (PANAS; Watson, Clark, & Tellegen, 1988) in the penultimate wave of data collection; although strictly the measurement was made prior to SA and self-rated health, the use of trait (dispositional) rather than state (situational) negative affect means it is likely to correlate well with negative affect at the time of the final wave.(Merz & Roesch, 2011) There was no evidence of any systematic differences between subgroups and we therefore present results based on all respondents combined.

We used logistic regression models to explore the possible interplay between SA dimensions by considering the impact on self-rated measures of each positive SA dimension adjusted for the others (e.g., no disease adjusted for no disability, good functioning and good social engagement; no disability adjusted for no disease, good functioning and good social engagement; and so on) to establish whether associations were being driven by a specific subgroup of SA dimensions. Finally, we considered whether a continuum offers a more realistic measure of SA than the more traditional, binary, success in all six dimensions, by calculating the prevalence of favorable self-rated health and satisfaction according to the number of positive SA dimensions.

In sensitivity analyses we recognized that SA dimension thresholds (i.e., the cut-offs for identifying positive vs negative SA dimensions) affect SA prevalence (McLaughlin et al., 2012) and examined the impact of varying thresholds in sensitivity analyses, replacing “best” tertiles of physical, cognitive, and social functioning with best quartiles, best quintiles, and above median measures; in all cases results were very similar.

## Results

The original 1932 and 1952 cohorts consisted of 1,551 and 1,444 respondents respectively, of whom 562 (36%) and 88 (6%) died during follow-up, 325 (21%) and 362 (25%) were not interviewed in Wave 5, and 181 (12%) and 108 (8%) had missing values for at least one SA dimension (most commonly cognitive function), leaving 483 and 886 in our analytical sample. Analyses by personality type were based on 449 and 775 respondents who were also interviewed in Wave 4. Respondents not included in analyses had lower SES and were more negative about their self-rated health and satisfaction. Mean (*SD*) age among 1932 and 1952 survivors at Wave 5 was 76.2 (0.6) and 57.1 (0.8), respectively.

Details of SA dimensions are presented in Table 1. The greater burden of chronic disease in the older cohort is clear, with only 58% free from disease versus 81% of the younger group. The prevalence of other positive dimensions in each cohort is similar by definition. The 1952 cohort had slightly more positive dimensions, reflecting the lower rate of chronic disease in this group. Notably, even in the younger cohort, very few respondents had all six dimensions positive, the conventional definition of SA, and around 80% of both cohorts had fewer than four.

Table 2 presents prevalences of favorable health and satisfaction and mean number of positive SA dimensions by age, gender, SES, and personality. Overall, respondents generally rated their health and satisfaction highly, although the prevalence varied by question, reflecting the different focus of each. For example, almost 69% of respondents considered their recent health to be good, rising to 76% for health *in the context of aging*, while 81% and 89% were happy with their health and life, respectively. The higher prevalences of self-rated satisfaction points to a group of respondents who rated their health badly but remained content, suggesting that many were accepting of and living successfully with poor health. Comparing the two age groups, older respondents reported less favorable recent health, consistent with the greater prevalence of chronic disease. However, strikingly, there was no marked difference in their self-rated health for age, or health and life satisfaction. Considering gender, SES and personality, women and, more markedly, respondents with manual SES or high negative affect were less positive about their self-rated health or satisfaction. In terms of the number of positive SA dimensions (Table 2, final column), the older cohort had fewer positive SA dimensions overall and the same was true of women and, most noticeably, manual SES respondents. However, in spite of differences in self-rated health and satisfaction, there was no difference in the mean number of positive SA dimensions in respondents with low versus high negative affect.

**Table 2.** Self-rated Health and Satisfaction and Number of Positive Successful Aging Dimensions by Age, Sex, Socioeconomic Status, and Personality^a^

	*N* (%) good vs poor self-rated recent general health^b^	*N* (%) good vs poor self-rated health for age^b^	*N* (%) positive vs negative self-rated health satisfaction^c^	*N* (%) positive vs negative self-rated life satisfaction^c^	Mean (95% CI) number of positive successful aging dimensions
Overall	938 (68.6)/1,368	1,042 (76.2)/1,368	1,102 (80.6)/1,368	1,211 (88.5)/1,368	3.3 (3.2, 3.4)
Age					
57	646 (73.0)/239	682 (77.1)/203	717 (81.0)/168	783 (88.4)/103	3.4 (3.3, 3.5)
76	292 (60.5)/191	360 (74.5)/123	385 (79.7)/98	428 (88.6)/55	3.2 (3.1, 3.3)
Gender					
Female	499 (67.3)/242	561 (75.7)/180	580 (78.3)/161	647 (87.3)/94	3.2 (3.1, 3.3)
Male	439 (70.0)/188	481 (76.7)/146	522 (83.3)/105	564 (89.8)/64	3.5 (3.4, 3.6)
Socioeconomic status					
Nonmanual	708 (73.6)/254	777 (80.8)/185	796 (82.7)/166	880 (91.4)/83	3.6 (3.5, 3.7)
Manual	225 (56.7)/172	260 (65.5)/137	299 (75.3)/98	323 (81.4)/74	2.6 (2.5, 2.8)
Negative affect^d^					
Below median	448 (73.6)/161	499 (81.9)/110	523 (85.9)/86	566 (92.9)/43	3.4 (3.3, 3.5)
Above median	399 (65.0)/215	444 (72.3)/170	475 (77.4)/139	523 (85.0)/92	3.3 (3.2, 3.4)

^a^Numbers differ due to missing responses to self-rated health and satisfaction.

^b^Good/excellent versus fair/poor health.

^c^Happy versus neutral or unhappy.

^d^Based on respondents who were also interviewed in Wave 4.

Differences (95% confidence interval) in the prevalence of favorable self-rated health and satisfaction in respondents with positive versus nonpositive SA dimensions are presented in Table 3. All positive SA dimensions were associated with better self-rated health and satisfaction and all confidence intervals excluded 0 (no difference). The largest differences in the prevalence of favorable self-rated health measures were those for disease and disability, with 17–27% more respondents free of disease or disability rating their health well. Differences in favorable self-rated health prevalence according to functioning and social engagement were smaller but broadly similar across SA dimensions (6–19% greater satisfaction in those with positive SA dimensions). The impact of SA on life satisfaction was fairly consistent across all dimensions (7–11% greater satisfaction). Associations were somewhat attenuated after adjustment for other SA dimensions (not shown). However, even after adjustment, marked increases remained in the number of respondents with positive versus negative SA dimensions who rated their health and satisfaction favorably. For example, after adjustment there was 13–20% greater satisfaction with health in respondents free of disease or disability, 3–14% greater satisfaction with health in respondents with good functioning and social engagement, and 4–8% greater life satisfaction in respondents with any positive SA dimension. This suggests that all SA dimensions contribute independently to the Rowe–Kahn model.

**Table 3.** Difference (95% Confidence Interval) in Prevalence of Good Self-rated Health or Positive Self-rated Satisfaction According to Individual Positive Versus Not Positive Successful Aging Dimensions (figure in square brackets is the prevalence of good self-rated health or positive self-rated satisfaction in respondents with nonpositive aging dimension)

	Good recent general health^a^	Good health for age^a^	Positive health satisfaction^b^	Positive life satisfaction^b^
Disease and disability				
No chronic disease	26.6 (20.8, 32.3); [49.2]	26.2 (20.7, 31.8); [57.0]	18.5 (13.3, 23.8); [67.0]	10.5 (6.1, 14.9); [80.8]
No disability	23.0 (18.3, 27.6); [58.6]	21.2 (17.0, 25.4); [67.0]	16.7 (12.8, 20.6); [73.3]	10.2 (7.0, 13.4); [84.0]
Functioning				
Good physical function	13.0 (8.0, 17.9); [64.2]	12.0 (7.6, 16.4); [72.1]	13.2 (9.3, 17.2); [76.1]	7.9 (4.7, 11.1); [85.6]
Good cognitive function	18.9 (14.2, 23.7); [62.2]	16.1 (11.8, 20.3); [70.8]	9.2 (5.0, 13.3); [77.5]	6.5 (3.2, 9.7); [86.3]
Social engagement				
Good interpersonal engagement	14.7 (9.6, 19.8); [64.6]	12.8 (8.3, 17.4); [72.7]	9.8 (5.6, 14.0); [77.9]	8.7 (5.6, 11.8); [86.1]
Good productive engagement	10.6 (5.0, 16.2); [66.3]	10.9 (6.0, 15.7); [73.8]	6.1 (1.4, 10.8); [79.2]	6.9 (3.5, 10.3); [87.0]

^a^Good/excellent versus fair/poor health.

^b^Happy versus neutral or unhappy.

**Figure 1.**
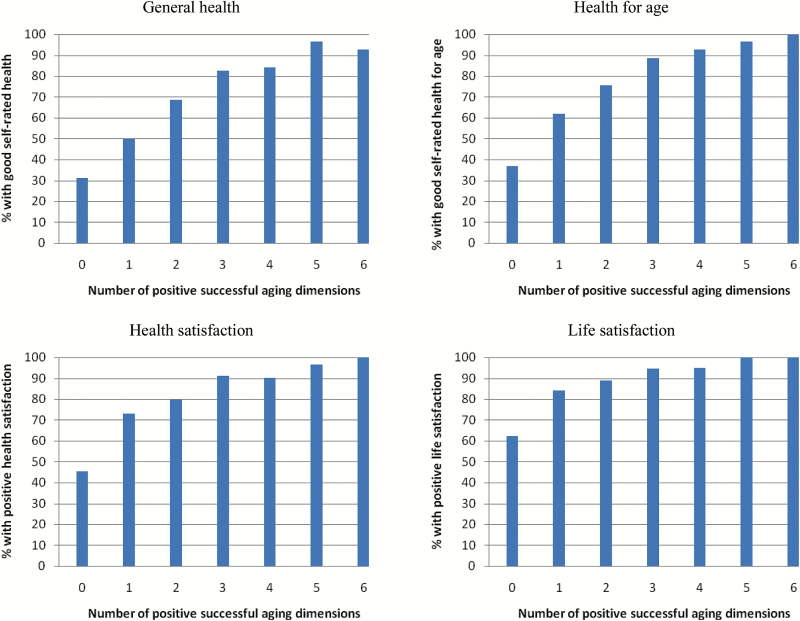
Prevalence of good self-rated health and satisfaction by number of positive successful aging dimensions.

The prevalence of good self-rated health and satisfaction increased consistently with increasing numbers of positive SA dimensions, with most or all respondents with six positive SA dimensions (conventionally successfully aging) rating their health and life favorably (Figure 1). However, over a third of respondents with no positive SA dimensions considered their health for age to be good, almost half were satisfied with their health, and almost two thirds were satisfied with their life in general. Strikingly, good self-rated health and satisfaction prevalence increased markedly with just two or three positive SA dimensions.

## Discussion

A straightforward, pragmatic approach to defining SA that is easily reproducible across different populations is highly important for policymakers and for measuring population health (Bloom et al., 2015; Commission of the European Communities, 2009; United Nations, 2002) The Rowe–Kahn model is easily constructed and expands on common biomedical models with the inclusion of social engagement. However, it has been criticised in the past for not including more nonclinical dimensions and, although this was not the intention (Kahn, 2002), for potentially setting a standard for SA below which individuals may be regarded as “failing” to age well. Previous comparisons of SA models (Bowling & Iliffe, 2006, 2011; Ferri et al., 2009; McLaughlin et al., 2012; Montross et al., 2006; Tate et al., 2003; von Faber et al., 2001) have generally used a subset of Rowe–Kahn dimensions or have simply compared prevalences of different SA measures rather than considering direct associations. However, strong positive associations have been reported between dichotomized SA, from the full Rowe–Kahn model, and self-reported wellbeing (Strawbridge et al., 2002) We observed similar associations with four established self-rated measures of health and life satisfaction, validating the Rowe–Kahn model more widely, and have expanded on previous work by comparing associations with individual SA dimensions, considering associations with the number of positive SA dimensions, and confirming that associations are consistent across age, gender, manual/nonmanual occupations, and personality.

The prevalence of our individual SA dimensions and self-rated measures indicate that, while many respondents were living with disease or disability, most reported feeling positive about their health *for age*, and the majority were happy with their health in particular and life in general. This paints an optimistic picture of aging in this population, consistent with the mantra “You’re only as old as you feel.” However, while self-rated health and satisfaction are important markers of SA, and may best capture respondents’ personal beliefs, they are unlikely, in isolation, to represent the complete aging experience, either at an individual or a population level. Additionally, self-rated measures of health and satisfaction are influenced by personal experiences, perceptions and expectations, which may be misleading when comparing across individuals. For example, in our cohorts, respondents with high and low negative affect had the same mean number of positive SA dimensions but, nonetheless, those with greater negative affect were markedly less likely to rate their health and satisfaction well. For policy purposes, and to make comparisons across individuals, we need a broad multi-dimensional definition of SA that incorporates both clinical and nonclinical dimensions but is not affected by prior expectations. In contrast to the results for self-rated health, the prevalence of positive Rowe–Kahn dimensions in our cohorts was independent of negative affect, suggesting that this model, while including an important nonclinical dimension, is not unduly influenced by individual preconceptions and expectations. Both objective and subjective SA measures have advantages and disadvantages in different circumstances. However, an objective measure, such as the Rowe–Kahn model, may be most appropriate in the context of identifying determinants of SA across different populations with a view to informing public health and economic policy and to identifying opportunities and interventions to improve SA.

Although the Rowe–Kahn model is widely recognized, many previous researchers have omitted dimensions, usually social functioning, counter to the importance placed on this aspect by older people (Cosco et al., 2013b; Phelan et al., 2004) In our cohorts, disease and disability were most markedly associated with self-rated health measures but, strikingly, differences in self-rated health prevalence according to social engagement were comparable to those for physical and cognitive functioning. Differences in the prevalence of life satisfaction were very similar for all SA dimensions, suggesting that the Rowe–Kahn model is a balanced measure in this context. Adjusted models confirm that social engagement and functioning associations are not simply an artefactual consequence of good health or vice versa. It is therefore important that researchers include all dimensions when utilising the Rowe–Kahn model and, in particular, that social engagement is not omitted, as this adds a unique nonclinical dimension. In addition, the strong associations between good social functioning and favorable self-rated health and satisfaction may have important implications for policy makers. For example, in addition to prevention of disease, disability and loss of functioning, there is potentially substantial value in policies that promote greater social engagement in older people such as lifelong learning, volunteerism, and participation in the arts.

Although we observed marked associations between positive Rowe–Kahn dimensions and positive reports of self-rated health and satisfaction, the Rowe–Kahn model undoubtedly presents a more pessimistic picture of SA than self-report in our population. SA is commonly defined as a dichotomy, with individuals succeeding or failing to meet a set of criteria and, in common with others (McLaughlin et al., 2010, 2012; Montross et al., 2006; Strawbridge et al., 2002) we found that, while only a small minority of respondents in both age-cohorts met all Rowe–Kahn criteria, most rated their health highly, particularly for age, and the majority were satisfied with their life. We therefore considered a less restrictive approach, based on the number of positive Rowe–Kahn dimensions, and observed increasingly good self-rated health and satisfaction with increasing numbers of positive SA dimensions. The idea of SA as a continuum has been proposed elsewhere (Bowling, 2007; Bowling & Iliffe, 2011; Young, Fan, et al., 2009; Young, Frick, et al., 2009) but has not been adopted. Our results suggest that this is a logical and informative approach that closely reflects older people’s opinions and expectations. Additionally, a continuum focuses on the *extent* of SA rather than a simple pass/fail. This is an important distinction as it acknowledges some of the natural consequences of aging (e.g., increasing disease/disability) while recognising the role of compensatory strategies (e.g., via social engagement) and may therefore be a more accurate representation of older people’s experience.

In spite of its strengths as an objective measure of SA, it is important to recognise that, even using a continuum, the Rowe–Kahn model does not completely correspond with older people’s views. This may reflect the limitations of self-rated measures, which, in isolation, do not necessarily incorporate all aspects of health (Au & Johnston, 2014) and, in the context of SA, may be unduly influenced by individual and cultural expectations, particularly among those in poor health (Sarkisian et al., 2002; Tate et al., 2003). Alternatively, while the Rowe–Kahn model incorporates both clinical and nonclinical dimensions, it does retain a biomedical emphasis, with two health versus one social dimension. An ideal measure might include more nonclinical dimensions such as self-efficacy, self-worth, resilience, and wellbeing. However, this would substantially increase the complexity and potentially limit the reproducibility of the measure, making it impractical in reality. In practice, the Rowe–Kahn model provides a pragmatic compromise between lay and clinical opinion and is based on factors that are fairly widely available in large population surveys, making it an efficient functional measure.

### Strengths and Limitations

Our analyses are based on two population cohorts, one representing the younger-old, aged around 57, who have not previously been studied in detail. In contrast to previous SA measure comparisons, we incorporated all Rowe–Kahn dimensions and considered these separately and in combination, using a continuum.

However, there are also limitations to be considered. Not all respondents from the original cohorts were included in our analyses; those excluded from the analyses were more likely to have manual SES and poor self-rated health, potentially limiting the generalizability of our results. There are no set thresholds for Rowe–Kahn dimensions and ours, like others, are relative to the study population, although results from analyses with more and less conservative definitions were very similar. In addition, although we have shown the value of considering the number of positive SA dimensions rather than a simple pass/fail measure, each individual SA dimension was based on a summary dichotomous variable. Future work might therefore explore the impact of combining the full range of data in each domain. Additionally, it may be interesting to consider SA trajectories across the life-course, to explore a wider age range, and to examine changes over time in different dimensions.

Finally, our analyses are based on two age-cohorts of men and women living in the West of Scotland in 2007/2008 and, as a result of geographic, cultural, and temporal variations (Jylha, 2009; Mitchell, 2005) it is possible that our self-rated health measures may differ from those in other populations. For example, a more stoic attitude to health has previously been reported in Scotland as compared with the rest of Great Britain.(Mitchell, 2005) In principle, this may have resulted in more positive reports of self-rated health and satisfaction in our population. However, similar discrepancies between lay and researcher-defined SA measures have been widely and consistently reported in a range of populations (McLaughlin et al., 2012; Montross et al., 2006; Strawbridge et al., 2002; von Faber et al., 2001; Young, Frick, et al., 2009) and we therefore consider it unlikely that the Scottish psyche is responsible in any significant way for these differences in the current analyses.

## Conclusion

The Rowe–Kahn model provides an easily reproducible objective measure of SA that is associated with subjective self-rated health and satisfaction, irrespective of age, gender, SES, and personality. Future work should consistently include all dimensions and consider SA as a continuum rather than an absolute state that very few older people achieve in reality. The Rowe–Kahn model is a functional measure and its consistent use in different populations, and how it relates to global measures of self-reported health, could further understanding of determinants of SA and inform policies aimed at increasing SA in aging populations.

##

**Key points**

- There is no consensus as to what constitutes “successful aging.”
- Traditional measures of successful aging may not represent the views of older people.
- The Rowe–Kahn successful aging model successfully captures older people’s views of their health and life satisfaction.

## Funding

The West of Scotland Twenty-07 Study is funded by the UK Medical Research Council (MC_A540_53462) and E.W. and F.P. are funded by the Medical Research Council (MC_UU_12017/7). M.B. is funded by the UK Economic and Social Research Council (Understanding Society waves 6–8) and the University of Essex.
